# Army News

**Published:** 1919-03

**Authors:** 


### ARMY NEWS

On March 2 the House of Representatives adopted the conference report in the general hospital bill, carrying an appropriation of $9,050,000. Among the army hospitals to be delivered to the Public Health Service for treatment of cases as the service deems advisable is that at Camp Logan, Houston, which hospital was substituted for the hospital at Camp MacArthur, Waco. Also the Public Health Service takes over army hospital No. 15 at Corpus Christi, under discretionary power of the Secretary of the Treasury, for which an appropriation of $150,000 is voted to purchase the building and site.

---

Dr. Tom Freundlich of Houston, Texas, who entered the service in July, 1917, has returned to civil life.

---

Soldiers honorably discharged since October 6, 1917, for disability incurred in line of duty and who again become incapacitated because of such disability are entitled to free hospital care under the provisions of the war insurance act, the War Department announced March 2nd.

---

Dr. R. M. Munroe has been given his honorable discharge from the Medical Corps of the army and has returned to Richmond. He will again be associated with Dr. O'Farrell and the partnership will be under the caption of O'Farrell & Munroe, as formerly.

---

Dr. Guy O. Shirey, prominent Fort Worth physician and a lieutenant colonel in the United States army, has returned to his home in Fort Worth after serving for almost two years with the American Expeditionary Forces in France.

---

Camp Bowie made the second best record of all camps in the country for having the smallest number of men to be sent to development battalions. First place went to Camp Taylor. This information was included in a statement issued at Washington by General March.

---

Dr. J. J. Delambse of Houston, Texas, has received his discharge, and after a rest and visit with friends in Louisiana, will resume his practice in Houston. He was in Belgium on the Flanders front in some of the fiercest fighting, having been wounded at Odenard.

---

Orders assigning fifty-two medical officers for duty with the American forces in Siberia were issued February 13 by the War Department. The party, including seven majors, nineteen captains and twenty-six lieutenants, will sail from San Francisco.

---

Lieutenant James F. Anderson, Medical Corps, stationed at the Great Lakes Naval Training Station Hospital, is in Fort Worth on a visit. He was a partner of his uncle, Dr. James Anderson, until a year ago, when he enlisted in the Navy. He will resume his practice in Fort Worth when he receives his discharge from the service.

---

Major James A. Hill, one of the prominent members of the medical profession in Houston and former president of the Harris County Medical Society, who has been in the service for more than a year, has obtained his discharge at the base hospital at Camp Logan, where he has been identified with the surgical department since coming to Camp Logan from Washington.

---

Lieutenant Hester B. Smith of the Army Medical Corps has received his discharge from the United States army and has returned to Dallas to resume his practice there.

---

Dr. W. W. Samuel has recently returned to Dallas since the demobilization of the unit to which he was attached as captain at Fort Sam Houston.

---

An election on $150,000 bonds for the erection for a memorial hospital to the Anderson County boys who died for their country will be held soon and more than 500 citizens signed the petition endorsing and pledging to vote for the issue. It is expected the bond issue will carry by a large vote.

---

The Board of Trustees of the State School for the Deaf has elected Dr. F. B. Shufford of Wolfe City, Hunt County, superintendent of the institution, and he qualified and entered upon the discharge of his duties March I.

